# Dyeing of advanced denim fabrics (blend of cotton/bicomponent polyester filaments) using different processes and artificial intelligence method

**DOI:** 10.1038/s41598-024-52189-y

**Published:** 2024-01-23

**Authors:** Marwa Souissi, Sabrine Chaouch, Ali Moussa, Hatem Dhaouadi

**Affiliations:** 1 Laboratory of Environmental Chemistry and Clean Processes, University of Monastir, Monastir, Tunisia; 2 National Engineering School of Monastir, University of Monastir, Monastir, Tunisia; 3 Textile Engineering Laboratory, University of Monastir, Monastir, Tunisia

**Keywords:** Chemical engineering, Chemical engineering, Environmental chemistry, Materials chemistry, Polymer chemistry, Process chemistry, Materials science

## Abstract

Denim clothes are the must-have items of clothing around the world. This kind of fabrics is evolving with the increasing consumer demand in order to keep its place as a versatile article. In this context, this paper contributes to the development and dyeing of a new blend fabric made of cotton fibers and bicomponent polyester filaments (PET/PTT). A comparative study between the mechanical and thermal properties of this fabric and conventional fabrics has confirmed the great interest to use bicomponent (PET/PTT) filaments in the manufacture of denim fabrics; these bicomponent filaments allow to give to wearer the desired elasticity and comfort. For dyeing (cotton/bicomponent polyester filaments) blend fabric, three different processes, using reactive and disperse dyes, were tested and analyzed. These dyeing processes are: two-baths/two-phases, one-bath/two-phases, and one-bath/one-phase processes. In addition, in order to obtain uniform shades between cotton fibers dyed with reactive dyes and bicomponent polyester filaments dyed with disperse dyes, an ant colony algorithm was elaborated to predict the optimal dye recipes. By observing obtained results, the developed algorithm is very effective; it allows to find the combination of reactive dyes necessary to achieve the same shade obtained by the disperse dyes with very small color differences between the two components and without having to make corrections mainly for the one-bath/two-phases process. Indeed, dyeing using the two processes (two baths/two phases and one bath/two phases) presents the best values of color yield (*K*/*S*) with almost similar results (*ΔE*_*CMC*(*2:1*)_ <  < 1). For the one-bath/one-phase process, it presents less significant results; We can observe *ΔE*_*CMC*(*2:1*)_ greater than 1 in certain shades. This is due to the strongly pH value (basic pH of 11) of reactive dyeing.

## Introduction

Nowadays, denim production is one of the world's major and constantly evolving textile industry sub-sectors. According to forecasts by the research firm of Prescient & Strategic, the global jeans market expected to post annual growth of 5.8% over the period 2018–2023^1^. To meet this ever-increasing demand, the denim industries have opted for new alternatives to remain competitive. They have moved towards more sustainable consumption by adopting the 4R strategy (Reduce, Repair, Reuse and Recycle).

Elastane is a filament that has very important elastic properties. This filament is widely used in the production of stretch denim articles. Indeed, the good elasticity of the elastane filament allows these garments to be close to the skin, therefore to be very comfortable and pleasant to wear. Thus, these clothing items are currently among the most sought after by the consumer. They represent a potential market for Denim manufacturers^1,2^. However, the presence of elastane in stretch denim articles poses a number of technical problems. Firstly, elastane is difficult filament to use. It must therefore to be covered by other textile materials such as cotton, lyocell, polyester, etc.^2^. In addition, it is highly sensitive to wet treatments such as pre-treatments, dyeing, finishing and heat treatments. In most cases, these treatments cause the degradation and loss of elasticity in stretch items, which is their most sought-after characteristic^3^.

Recently, bicomponent filaments (PET/PTT) have been used in the manufacture of various textile fabrics to provide thermal comfort thanks to their elasticity and their elastic recovery^4–13^. These filaments are an excellent alternative for overcoming the technical difficulties associated with the use of elastane filaments^5^. They are composed of two polymers: polyethylene terephthalate (PET) and polytrimethylene terephthalate (PTT). These two filaments are adjacent, arranged side by side and extruded from the same spinneret^4–13^.

In previous studies^14–21^, these bicomponent filaments are characterized in terms of their mechanical, chemical and physical properties. Obtained results showed their excellent elastic and recovery elasticity and confirm their ability to replace elastane filaments. A recent study of the stability of bicomponent filaments after heat treatments highlighted the conservation of the desired mechanical properties of these filaments^18^.

Consequently, in this work, a new denim item was produced. Warp and weft yarns are composed of 100% cotton fibers and 100% bicomponent (PET/PTT) filaments, respectively. However, a problem arises concerning the dyeing of this cotton/bicomponent polyesters blend fabric. Indeed, 100% cotton warp yarns are dyed with reactive dyes while polyesters filaments are dyed with disperse dyes. The challenge is to formulate and reproduce the same color for warp and weft yarns to have a final article with uniform shade.

In the literature, several studies have focused on color recipes prediction, either using colorimetric and spectrophotometric methods^22–25^ or using artificial intelligence techniques^26–30^. Recently, Chaouch et al. developed a new genetic algorithm for color recipe prediction of cotton fibers using reactive and direct dyestuffs^31^. The studied parameters of the algorithm were determined using a full factorial experimental design. The evaluation of the efficiency of the algorithm was proven and the obtained recipes showed good colorimetric correspondence with the target colors; all color difference ΔE_CMC(2:1)_ values are less than 1. The same authors developed a second method based on ant colony algorithm^32,33^.

The latter seems to be a promising method for solving the problem of color recipe prediction. Obtained results were very encouraging; theoretical values of ∆E_CMC(2:1)_ between the target colors and those proposed by the ant colony algorithm do not exceed 0.7. These results reveal the excellent conformity of the predicted concentrations to the desired shades. More recently, a third study focused on comparing these two color prediction algorithms, genetic and ant colony algorithms, in order to determine which best optimizes the color formulation step^34^. Based on this study, the ant colony algorithm seems to be the most efficient algorithm in predicting dyeing recipes of cotton fibers with reactive dyes.

Therefore, in this current study, this evolutionary ant colony algorithm was used to determine the appropriate recipes for dyeing our innovative fabric composed of a blend of cotton/bicomponent polyester filaments. First, we presented in the first step the manufacturing parameters of the new (cotton/bicomponent polyester filaments) blend fabric. Then a comparison study between its mechanical and thermal comfort properties with a conventional denim fabric (100% cotton) and a blend fabric (cotton/PET filaments) was established. For the dyeing of this new fabric, clean dyeing was performed using different processes: two-baths/two-phases, one-bath/two-phases, and one-bath/one-phase. The ant colony algorithm was then applied to predict the appropriate recipes in order to find uniform shades.

## Materials and methods

### Textile supports

The textile supports used in this study are: 100% cotton fabric used as a reference (F1), cotton/PET blend fabric (F2), and cotton/bicomponent (PET/PTT) filaments blend fabric (F3). All these fabrics are produced using a Sulzer-type projectile weaving. The blend fabrics are made from the interlacing of 100% cotton warp threads and 100% polyester weft threads. All the fabrics produced using 2/2 twill weave. The number of weft threads per centimeter is equal to 17. The main characteristics of the fabrics obtained are presented in Table 1.

**Table 1:** Main characteristics of studied fabrics.

Fabric code	Warp yarn composition	Weft yarn composition	Warp count (tex)	Weft count (tex)	Fabric thickness (mm)	Fabric weight (g m^2^)
F1	100% cotton	100% cotton	25	33	0.803 ± 0.005	395 ± 0.038
F2	100% cotton	100% PET filaments	25	25	0.677 ± 0.006	298 ± 0.020
F3	100% cotton	Bicomponent (60% PET/40% PTT) filaments	25	33	0.760 ± 0.003	375 ± 0.035

### Characterization of textile supports

#### Mechanical tests

The mechanical properties of fabrics were made according to the standard method ISO 13934-1 and using a dynamometer type Lloyd instruments LR5K (UK). The dimensions of tested samples are equal to 5 cm × 25 cm. Each measurement was repeated five times and carried out in both warp and weft directions of the fabric. The test speed is maintained at 100 mm min^−1^.

#### Moisture management test

According to the AATCC TEST METHOD 195 (2011), moisture management behaviours of studied fabrics were evaluated using the Moisture Management Tester (MMT) type M290 (SDL ATLAS, USA). Tested fabrics are cut in the form of a square with a side equal to 8 cm, the pumping time of the saline solution is equal to 20 s and the duration of each test is 120 s. Each test was repeated three times.

### Dyeing recipes

**Figure 1:**
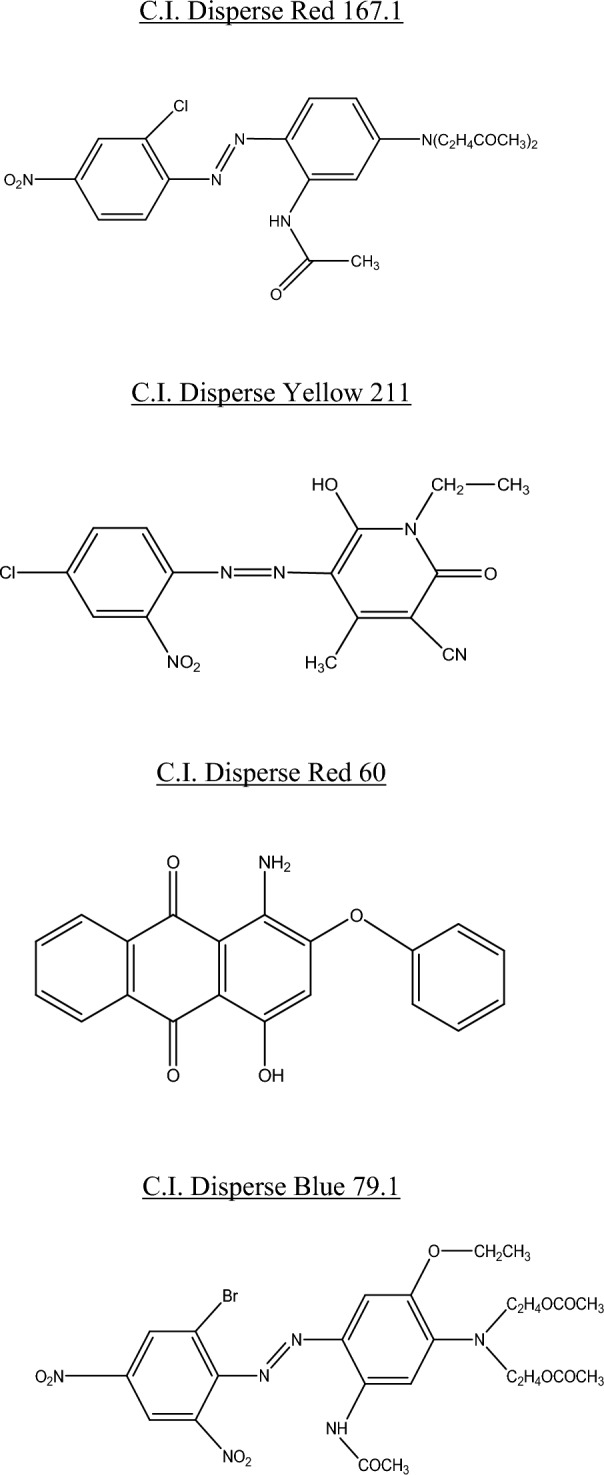
Chemical structures of disperse dyes.

**Figure 2:**
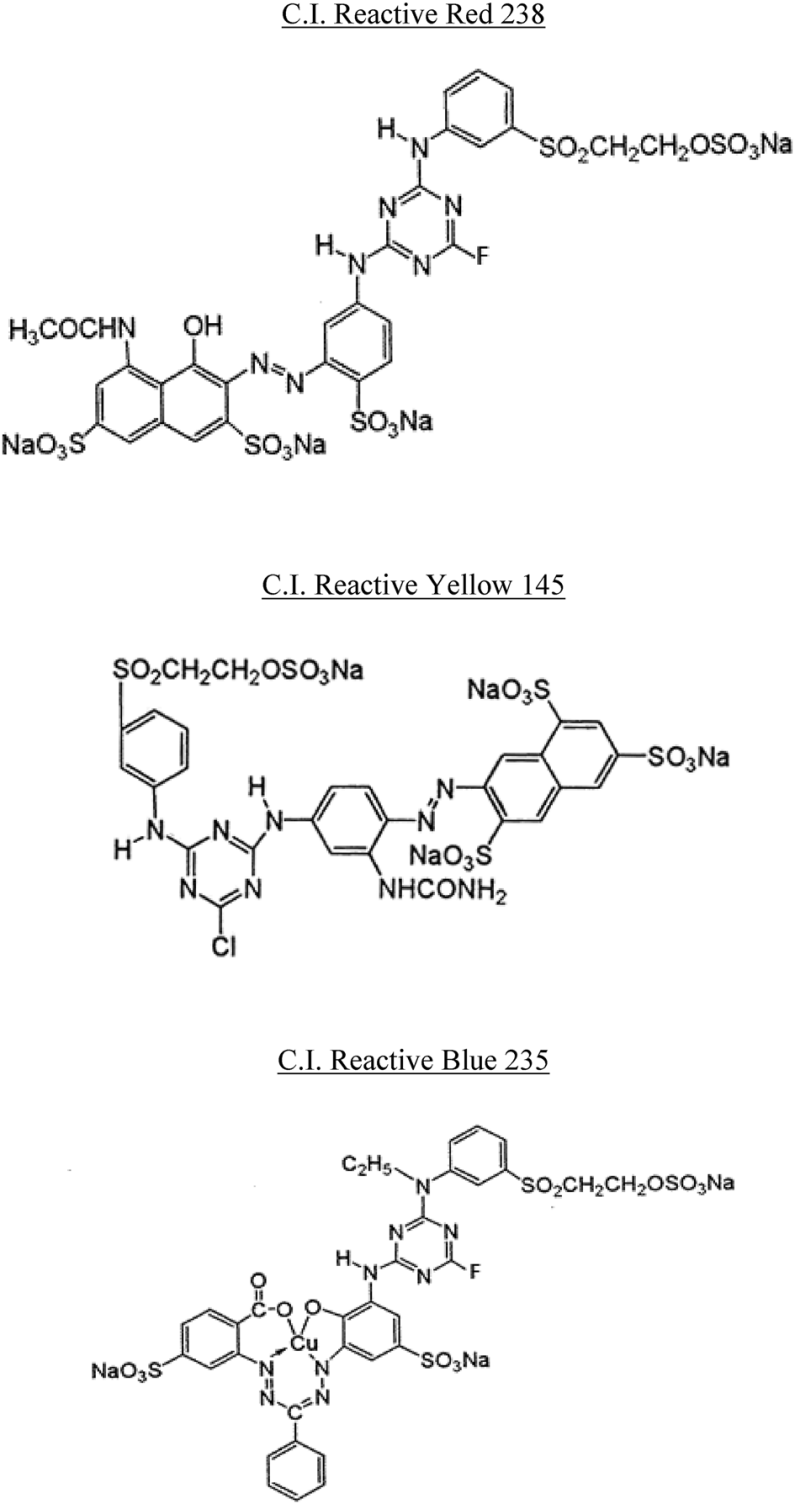
Chemical structures of reactive dyes.

In this study, our blend fabrics (cotton/bicomponent polyester filaments) were dyed using reactive and disperse dyes. The cellulosic part was dyed using three reactive dyes, namely: C.I. Reactive Red 238, C.I. Reactive Yellow 145 and C.I. Reactive Blue 235. However, the dyeing of the polyester part was carried out using four disperse dyes, namely: C.I. disperse Red 60, C.I. disperse Yellow 211, C.I. disperse Red 167.1 and C.I. Disperse Blue 179.1. All reactive and disperse dyes were purchased from Huntsman (Switzerland) and used without purification. Table 2 presents the main characteristics of used dyes. Their chemical structures are shown in Figs. 1 and 2. The liquor ratio is equal to 1:10. For dyeing cotton with reactive dyes, the following auxiliary products were used: CHTT-MRS (wetting agent), sodium chloride and caustic soda. The added quantities of auxiliaries depended on the desired shades. Dyeing of the polyester part required only the use of p-vanillin carrier with a concentration equal to 0.04 mol/L.

**Table 2:** Main characteristics of studied dyes.

Range of dyes	Color index	Chemical formula	Molecular weight (g mol^−1^)
Disperse dyes	CI Disperse Red 167.1	C_22_H_24_ClN_5_O_5_	473.15
	CI Disperse Yellow 211	C_15_H_12_ClN_5_O_4_	361.74
	CI Disperse Red 60	C_20_H_13_NO_4_	331.08
	CI Disperse Blue 79.1	C_24_H_27_BrN_6_O_10_	639.41
Reactive dyes	CI Reactive Red 238	C_29_H_21_FN_8_O_17_S_5_Na_4_	1024.80
	CI Reactive Yellow 145	C_28_H_20_ClN_9_O_16_S_5_Na_4_	1026.22
	CI Reactive Blue 235	C_33_H_24_FCuN_9_O_15_S_4_Na_3_	1066.37

### Dyeing procedure

To dye (cotton/bicomponent polyester filaments) blend fabric, three dyeing processes were used, namely: two-baths/two-phases process, one-bath/two-phases process, and one-bath/one-phase process. These processes are inspired by those used for dyeing (cotton/PET) blend fabrics. The dyeing of the polyester part, i.e. bicomponent (PET/PTT) filaments, was carried out using a clean and ecological process already developed in our previous works^15,17,21^.

All pre-treatments, dyeing and post-treatments were carried out in a laboratory dyeing machine, an Ahiba Nuance Top Speed (Datacolor International Company, USA).

#### Two-baths/two-phases dyeing process

**Figure 3:**
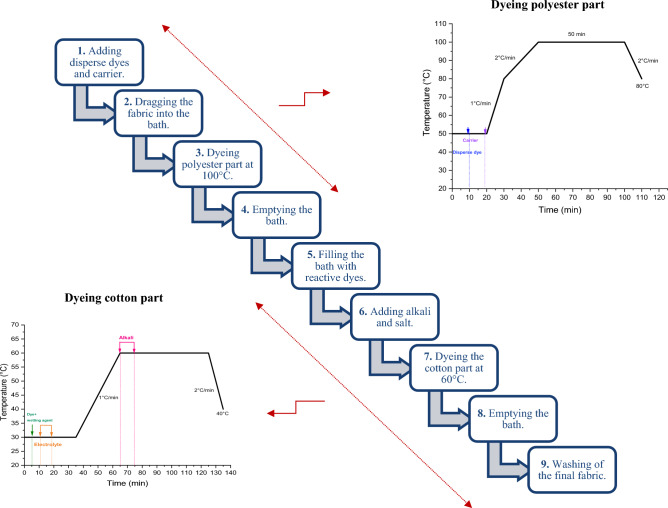
Dyeing (cotton/bicomponent polyesters filaments) fabric using two-baths/two-phases dyeing process.

This process is one of the most commonly used processes in the textile industry^25,26^. It involves fixing reactive and disperse dyes using two different baths. The steps and the thermal curves of this dyeing process are mentioned in Fig. 3.

#### One-bath/two-phases dyeing process

**Figure 4:**
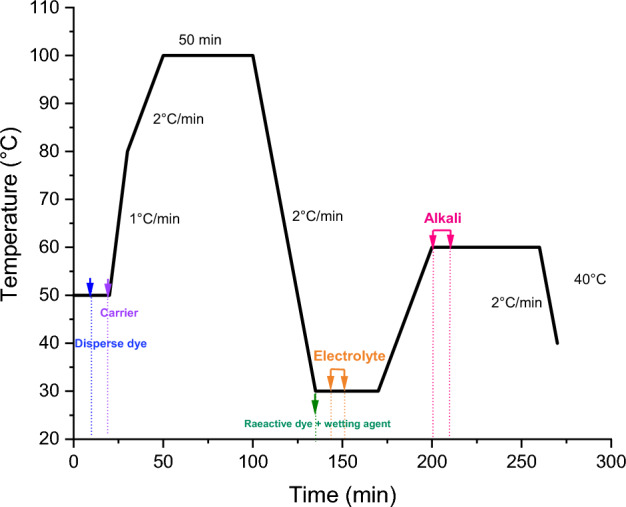
Dyeing (cotton/bicomponent polyesters filaments) fabric using one-bath/two-phases process.

This dyeing process, shown in Fig. 4, first dyes the polyester part with disperse dyes at 100 °C with the addition of p-vanillin. The bath was then cooled to 30 °C. At this level, the reactive dye and the wetting agent and the electrolyte were added. The reactive dyes were then fixed for 40 min. A pH of 11 was reached by adding alkali. After dyeing, a rinse at 50 °C, neutralization with acetic acid at 50 °C, soaping at 95 °C and another cold rinse were carried out to remove the dye persisting on the fabric surface.

#### One-bath dyeing process

**Figure 5:**
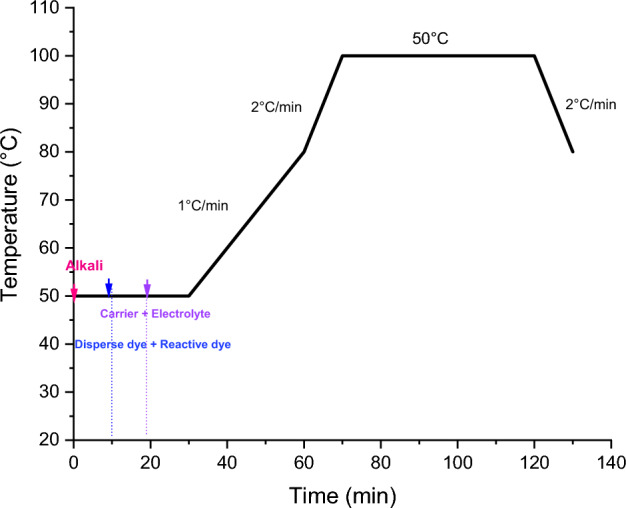
Dyeing (cotton/bicomponent polyesters filaments) fabric using one-bath/one-phase process.

To dye the (cotton/bicomponent polyester filaments) blend fabric, a single-bath dyeing process was also used (Fig. 5). At the start of this process, the alkali was added to adjust pH at 11. The two classes of dyes (reactive and disperse) were then introduced followed by the electrolyte and the carrier. In this case, the polyester part and the cotton part was dyed at 100 °C at the same time in the same bath. After 40 min, samples underwent several hot and cold rinses.

### Color measurement

All colorimetric measurements were performed using a Spectraflash 600 Plus type spectrocolorimeter (Datacolor International, USA) with the following measuring conditions: spectra from 400 to 700 nm at 10 nm intervals, illuminant D65, and 10° standard observer. In order to assure maximum opacity, each sample was presented at four layers; and to reduce errors in reflectance measurements, three color measurements were made per sample in multiple locations.

The Kubelka–Munk theory linking the color strength (*K/S*) to the reflectance* R* is presented by the following formula^37^:

1 $$\frac{K}{S}=\frac{{\left(1-R\right)}^{2}}{2R}-\frac{{\left(1-{R}_{0}\right)}^{2}}{2{R}_{0}}$$

where *K*, *S,* and *R* are respectively the absorption coefficient, the light scattering coefficient and the reflectance of the dyed sample. *R*_*0*_ is the reflectance of the undyed sample.

### Description of the ant colony algorithm

Ant colony algorithm is an artificial intelligence technique that dates back to the 1990s^38^. It has been used in various fields such as vehicle routing^39^**,** graph coloring^40^ bioinformatics problems^41^, etc. This technique is based on the attitude of ants during their search for food. Indeed, during this search, ants cross random paths while leaving behind a chemical substance called pheromones. Thanks to this substance, the other ants will be guided towards the source of food. The amount of pheromones smelled depends on the quality and quantity of food found. This indirect communication method is very effective and allows ants to find the shortest path to food.

In the literature, Chaouch et al.^32,33^ were the first authors to introduce ant colony optimization (ACO) for color formulation. They proposed an algorithm of color matching allowing to minimize the color deviations between the standard colors and colors obtained by the proposed recipes. This algorithm was developed for dyeing cotton samples using reactive and direct dyes. We therefore applied this artificial intelligence technique to formulate the color recipes of our new (cotton/bicomponent (PET/PTT) filaments) blend fabric. Indeed, after dyeing the bicomponent filaments with disperse dyes, we used the ant colony algorithm to predict best reactive dye recipe to match the color obtained with disperse dyes (considered as standard or target color).

The developed algorithm assumes:

- First, the food source is the desired standard color (obtained by disperse dyes);
- Second, the paths explored by ants searching represent the various shades of available reactive dyes used in mixtures;
- Third, the shortest path, lied the food to the ant nest, corresponds to the best reactive recipe that optimizes color deviations between the obtained color (reproduced by this best reactive recipe) and the standard color (obtained by disperse dyes).

In our case, the objective is to obtain the same color for the two components of the fabric, i.e. the bicomponent filaments and the cotton fibers. The objective function of the algorithm is then the minimization of the CMC(2:1) color deviations [*ΔE*_*CMC*(*2:1*)_] between colors obtained on bicomponent filaments dyed with disperse dyes and cotton fibers dyed with reactive dyes.

To match the color obtained by disperse dyes, considered as standard color, the developed algorithm works as follows: each ant selects a concentration value between 0 and 4% for each dye from the available reactive dyes. For the first iteration, this choice is made randomly, but for subsequent iterations, it is based on defined probabilities. The reactive color recipe formed by the chosen concentrations represents a path or solution. When this recipe is applied to dye cotton fibers, it produces a color that may be more or less similar to the standard color. The most appropriate recipe of reactive dyes is the one that minimizes the shade deviations between the proposed color and the standard one. To ensure successful color formulation, the ACO algorithm must propose the best reactive dyeing recipe that results in a standard color with a [*ΔE*_*CMC(2:1)*_] deviation that does not exceed 1. If multiple recipes are possible, we select the recipe that minimizes the [*ΔE*_*CMC(2:1)*_] deviation.

At each iteration *t*, the choice of the concentrations is done randomly, based on the probability value *P*_*ij*_:

2 $${P}_{ij}\left(t\right)= \frac{{\tau }_{ij}^{\alpha }(t).{\eta }_{ij}^{\beta }}{\sum_{l\in {N}_{i}^{k}}{\tau }_{il}^{\alpha }(t).{\eta }_{il}^{\beta }}$$

where: ${P}_{ij}\left(t\right)$ is the probability value to select the concentration index *j* of the succeeding dyestuff afterward choosing the concentration index *i* of the present dyestuff; τ_ij_ is the quantity of pheromones deposed between the concentration index *i* of the present dyestuff and the concentration index *j* of the succeeding dyestuff; ${\eta }_{ij}= \frac{1}{{\Delta E}_{CMC(2:1)}}$ is the visibility value between concentrations index *i* and *j*; ${N}_{i}^{k}$ is the concentrations range of dyestuffs not yet chosen at the instant *t*; *α* and *β* are the variables supervising the relative importance between the rate of pheromones and visibility, respectively.

The pheromone matrix is updated locally as follows:

3 $${\tau }_{ij}\left(t\right)= \rho .{\tau }_{ij}\left(t-1\right)+\Delta {\tau }_{ij}$$

where: *ρ* is the evaporation rate of pheromones; $\Delta {\tau }_{ij}= \frac{{C}_{local}}{{\Delta E}_{CMC(2:1)}}$ is the quantity of pheromones, called *local pheromone trace*, deposed by the different ants after their passageways at the iteration index *t*; *C*_*local*_ is a positive constant fixed at the beginning of the algorithm.

After each iteration, the global update of the pheromone matrix aims to provide a greater quantity of pheromones to the best solution; it is applied to the best path, followed by the ant index *k’*, using the following formula:

4 $${\tau }_{ij}\left(t,k^{\prime}\right)= {\tau }_{ij}\left(t,k^{\prime}\right)+\Delta {\tau ^{\prime}}_{ij}$$

where: $\Delta {\tau ^{\prime}}_{ij}= \frac{{C}_{global}}{{\Delta E}_{CMC(2:1)}}$ is the *global trace pheromone* adding to the best path (followed by ant index *k’*) in order to promote the best recipe found during the iteration index *t*; *C*_*global*_ is a positive constant fixed at the beginning of the algorithm.

**Figure 6:**
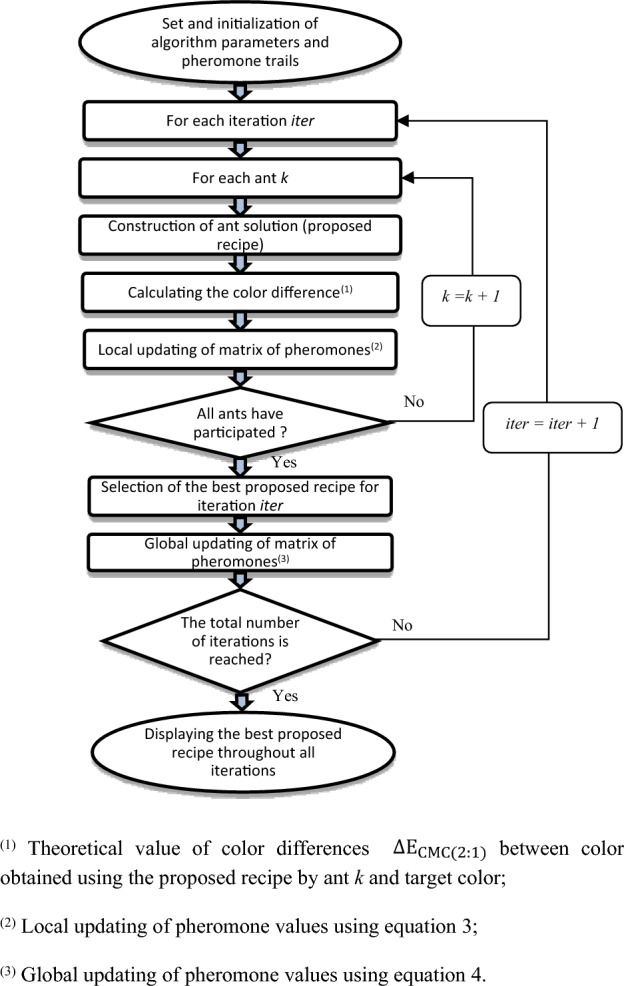
Flow chart of the ant colony algorithm.

The algorithm procedure is composed by four principal steps (Fig. 6):

- Step 1: setting of algorithm parameters and pheromone trails;
- Step 2: construction of ant solutions (proposed recipes);
- Step 3: updating of the matrix of pheromones;
- Step 4: repeating steps number 2 and 3 until the end conditions are reached (i.e. all ants are chosen and the total number of iterations is reached).

For the dyeing of our blend fabric (cotton/bicomponent polyester filaments), the algorithm predicts the best recipe among all available reactive dyes for dyeing cotton fibers, to achieve the same shade as that of the bicomponent polyester filaments. Then, depending on the range of dyes chosen for each component, we will opt for the appropriate process (one-bath/one-phase, one-bath/two-phase or two-bath/two-phase dyeing process).

## Results and discussions

### Mechanical properties of studied fabrics

To highlight the excellent mechanical properties of our fabric (F3) made from a blend of bicomponent filaments (cotton / (PET/PTT)), a comparative study was carried out with two conventional fabrics: fabric (F1) made from 100% cotton and fabric (F2) based on a cotton/PET blend. The mechanical properties of these three fabrics are presented in Table 3. By observing the obtained results, it can be concluded that the fabric (F1) is much more stiffer than fabrics (F2) and (F3) whose weft yarns are in classic polyester (100% PET) and in bicomponent filaments (60% PET, 40% PTT), respectively. These obtained results are due to the fact that polyester threads are much more resistant than cotton fibers. In addition, the fabric (F3) has an elongation percentage equal to 50.71%. This is a very high value compared to the two others fabrics. These excellent mechanical performances showed that geometry shape of filaments has a great effect on their elastic recovery. The bicomponent filaments have a high value of recovery, thanks to the arrangement side by side of the two polymers PET and PTT. This good elasticity enables the blended fabric (cotton/bicomponent filaments (60% PET, 40% PTT)) to adapt to the wearer's body, to be soft and comfortable.

**Table 3:** Mechanical properties of studied fabrics.

Fabric	Composition		Elongation (%)		Resistance (MPa)		Rigidity (N/m)		Young's modulus (MPa)	
	Warp yarn	Weft yarn	Warp direction	Weft direction	Warp direction	Weft direction	Warp direction	Weft direction	Warp direction	Weft direction
F1	100% cotton	100% cotton	27.14 ± 0.42	26.20 ± 0.23	39.99 ± 0.42	42.89 ± 0.20	72,954 ± 0.43	41,189 ± 0.34	432 ± 0.31	342 ± 0.24
F2	100% cotton	100% PET	24.68 ± 0.39	19.32 ± 0.55	45.22 ± 0.28	12.65 ± 0.22	74,968 ± 0.35	18,092 ± 0.36	444 ± 0.24	107 ± 0.15
F3	100% cotton	60% PET/40% PTT	27.3 ± 0.24	50.71 ± 0.31	45.49 ± 0.22	27.34 ± 0.43	79,089 ± 0.22	18,831 ± 0.43	422 ± 0.20	98 ± 0.39

*F1* fabric made of 100% cotton, *F2* fabric made of (cotton/PET) blend, *F3* fabric made of (cotton/(60% PET/40% PTT) bicomponent filaments) blend.

### Thermal comfort properties of studied fabrics

According to the AATCC TEST METHOD 195 (2011), the ability to evacuate sweat of studied fabrics, in particular the fabric made of a cotton/bicomponent polyester filaments blend, was evaluated. Table 4 illustrates results of this analysis. From obtained results, it can be seen that humidification time (sec), absorption rate (%.s), maximum moisture diffusion radius (mm), and moisture diffusion rate (mm/s) values of our fabric are all good. More importantly, the percentage of moisture transport capacity (%) values are excellent and the overall moisture management capability (OMMC) value is also good. It can be concluded that the manufacture of this fabric, on the one hand, remedies all the problems caused by the use of elastane filaments and, on the other, produces a fabric with good, even excellent, thermal comfort properties. This may encourage the use of denim fabrics that offer the user both good elasticity and elastic recovery, as well as good thermal comfort properties.

**Table 4:** Thermal comfort properties of (cotton/bicomponent polyester filaments) blend fabric.

Parameters	Outer face	Internal face	Grade*	Performance
Humidification time (sec)	9.02 ± 0.03	10.4 ± 0.05	3	Good
Absorption rate (%.s)	21.48 ± 0.20	33.08 ± 0.15	3	Good
Maximum moisture diffusion radius (mm)	11 ± 0.14	14 ± 0.18	3	Good
Moisture diffusion rate (mm/s)	1.24 ± 0.01	1.31 ± 0.07	3	Good
Moisture transport capacity (%)	425.4 ± 0.10	180.36 ± 0.13	5	Excellent
Overall moisture management capability (OMMC)	0.53 ± 0.22		3	Good

*Grade: (1) low; (2) medium; (3) good; (4) very good; (5) excellent.

**Figure 7:**
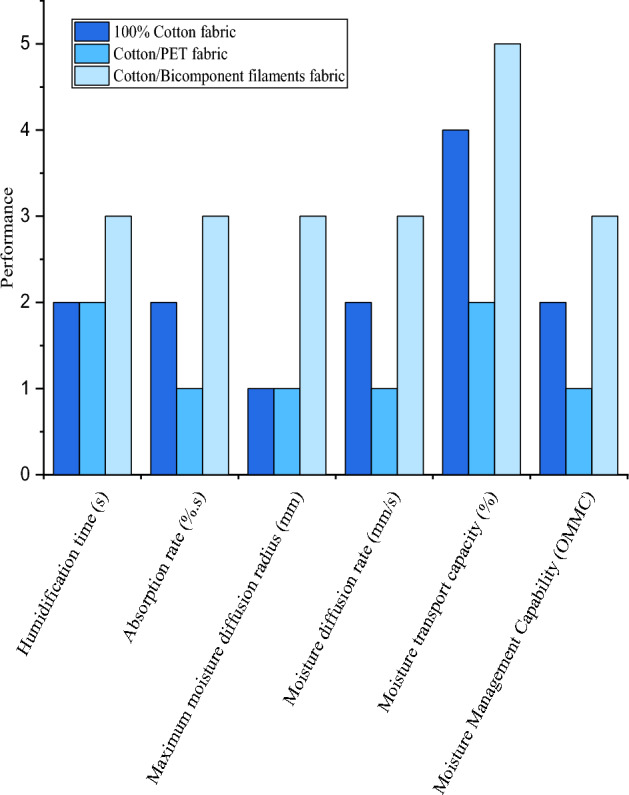
Thermal comfort properties of studied fabrics.

In addition, a comparative study between the different fabrics developed was carried out. Figure 7 shows that the fabric containing bicomponent polyester filaments offers the best performance, followed by the 100% cotton fabric and the cotton/PET blended fabric. Indeed, the contact surface is larger than that of conventional fibers with a rounded cross-section; therefore, the moisture will diffuse more easily and more quickly by capillary action.

This confirms the great potential for the use of bicomponent (PET/PTT) filaments in various textile articles, in particular denim articles mixed with cotton.

The big challenge is to dye these innovative fabrics correctly, and above all to guarantee the same shades for the two materials used: cotton and bicomponent filaments (PET/PTT).

### Analysis of dyeing performances

In order to obtain the same color on the different components of the fabric, a dye recipe prediction algorithm was developed and applied. This algorithm, based on ant colony optimization technique, must predict and propose the most appropriate recipe of reactive dyes in order to match the shade obtained by disperse dyes. The shade used for disperse dyes is 1.2% of each one. So, the ant colony algorithm was applied to determine the equivalent recipe to dye the cotton part and obtain the same shade as that of the polyester part.

The predicted recipes are presented in Table 5. These proposed recipes were used for the three processes (two-bath/two-phase, one-bath/two-phase and one-bath/one-phase) for dyeing the cotton/bicomponent filament blend. The (*K/S*) values obtained as well as the CIELab colorimetric coordinates of the dyed cotton/bicomponent filament (60% PET, 40% PTT) blend fabrics are given in Table 6.

**Table 5:** Dyeing recipes for cotton/bicomponent filament blend fabrics.

Shades	Dyeing recipes for polyester part (actual recipe)		Predicted dyeing recipes for cotton part (proposed by our ant colony algorithm)			
	Disperse dyes	Carrier (g/L)	Reactive dyes	Wetting agent (mL/L)	Electrolyte (g/L)	Alkali (mL/L)
Shade 1	1.20% C.I. Disperse Red 60	6.00	2.25% C.I. Reactive Red 238 + 0.30% C.I. Reactive Yellow 145	3.00	70.00	2.90
Shade 2	1.20% C.I. Disperse Yellow 211	6.00	2.00% C.I. Reactive Yellow 145	3.00	63.90	2.66
Shade 3	1.20% C.I. Disperse Red 167.1	6.00	4.00% C.I. Reactive Red 238 + 0.80% C.I. Reactive Yellow 145 + 0.05% C.I. Reactive Blue 235	3.00	99.50	3.74
Shade 4	1.20% C.I. Disperse Blue 179.1	6.00	2.50% C.I. Reactive Blue 235	3.00	70.00	2.90

**Table 6:** *(K/S)* values and color coordinates of blend fabrics (cotton/bicomponent (PET,PTT) filament) dyed using the different dyeing processes.

Shades	Dyeing process	(*K/S*)	Color coordinates (D65/10°)					Color differences					
			L*	a*	b*	C*	*h* (°)	ΔL*	Δa*	Δb*	ΔC*	Δ*H*	Δ*E*_*CMC(2:1)*_
Shade 1	Two baths/two phases	19.50 ± 0.15	26.36	33.93	6.56	34.56	10.95	–	–	–	–	–	–
	One bath/two phases	19.35 ± 0.25	26.43	34.02	6.58	34.65	10.95	−0.07	−0.09	−0.02	−0.09	0.00	0.059
	One bath/one phase	19.05 ± 0.34	26.76	37.09	7.04	37.75	10.75	−0.37	−3.16	−0.48	−3.19	0.20	1.510
Shade 2	Two baths/two phases	14.21 ± 0.41	59.32	20.55	35.11	40.68	59.69	–	–	–	–	–	–
	One bath/two phases	14.30 ± 0.17	59.21	20.52	35.09	40.65	59.71	0.11	0.03	0.02	0.03	−0.02	0.042
	One bath/one phase	14.15 ± 0.08	59.44	20.59	35.13	40.72	59.66	−0.23	−0.04	−0.02	−0.04	0.03	0.049
Shade 3	Two baths/two phases	18.62 ± 0.34	28.28	41.11	3.74	41.28	5.20	–	–	–	–	–	–
	One bath/two phases	18.69 ± 0.23	28.07	41.15	3.89	41.33	5.40	−0.07	−0.04	−0.15	−0.05	−0.20	0.175
	One bath/one phase	17.96 ± 0.15	28.41	49.15	4.09	49.32	4.76	0.66	−8.04	−0.35	−8.04	0.44	3.437
Shade 4	Two baths/two phases	12.53 ± 0.20	22.07	2.95	−15.77	16.04	79.44	–	–	–	–	–	–
	One bath/two phases	12.49 ± 0.32	22.13	2.97	−15.69	15.97	79.32	0.04	−0.02	−0.08	0.07	−0.12	0.128
	One bath/one phase	11.53 ± 0.36	23.36	3.42	−16.89	17.23	78.59	1.00	−0.47	1.12	−1.19	−0.85	1.442

By observing obtained results, it is clear that (*K/S*) values and CIELab coordinates of the samples dyed using the three different processes are very close. These results confirm the effectiveness of the developed ant colony algorithm which enables us to find the combination of reactive dyes needed to obtain the same shade as that obtained with the disperse dyes without having to make any corrections. In addition, the use of this algorithm offers the possibility to remedy wastage during the use of dyes and to reduce the quantity of water used during color corrections.

It should be noted that dyeing using the first two processes (two baths/two phases and one bath/two phases) presents the best values of color yield (*K/S*) with almost similar results (color differences Δ*E*_*CMC(2:1*)_ <  < 1). Indeed, if the first involves dyeing the polyester part and then emptying the dye pots before proceeding to dye the cotton, the latter involves keeping the same dye bath after dyeing the polyester part and then incorporating all the products required for reactive dyeing. It can therefore be concluded that the disperse and reactive dyes used in this study are compatible and during their use in both processes, no degradation of either dye was observed. The levelness of dyed fabrics is also good.

For the third dyeing process, one-bath/one-phase, it presents less significant results; We can observe (Δ*E*_*CMC(2:1)*_) color differences greater than 1 in shades 1, 3 and 4. This is due to the strongly pH value (basic pH of 11) of reactive dyeing. Indeed, several previous studies have shown that disperse dyes do not tolerate high values of basic pH, and in such cases, color deviations would be possible^35,36^.

Furthermore, we observed that the use of p-vanillin did not cause problems in the dyeing of the reactive part even when using the two processes, one bath/two phases and one bath/one phase, where the dyebath was not emptied or changed during the dyeing of the blend fabric (cotton/bicomponent (PET/PTT) filament). In this way, the bicomponent (PET/PTT) filaments were dyed using a clean and economical process in terms of temperature (100 °C) and the dyeing process for cotton/polyester blend fabrics can also be economical in terms of water consumption by keeping the same dye bath and therefore using a one-bath/two-phases process.

These excellent dyeing results could encourage the manufacture of articles containing bicomponent (PET, PTT) filaments and their incorporation into denim fabrics to give the wearer the desired elasticity and comfort. They also allow the use of an economical and ecological dyeing process.

## Conclusion

This paper contributed to the development of a new fabric made of (cotton/ bicomponent polyester filament) blend. A comparative study between the mechanical and comfort thermal properties of this new fabric and those made of 100% cotton and (cotton/PET) blend has confirmed the interest of the developed fabric. This encourages the manufacture of articles containing bicomponent (PET, PTT) filaments and their incorporation into denim fabrics in order to give the wearer the desired elasticity and the desired comfort.

Moreover, this study proposed a suitable process for dyeing this kind of fabrics and evaluated its performance. Faithful to our principles of green chemistry, the one-bath/two-phases process seems to be the most appropriate process for dyeing (cotton/bicomponent (PET, PTT) filament) blend fabric.

In addition, the use of the ant colony algorithm, developed in this study, has been very effective in predicting the right dye recipes and in formulating colors without having to make multiple corrections and while minimizing the amount of dyes and water. Obtained results proved that this algorithm is a very powerful tool in the prediction of dye recipes and the formulation of shades for blend fabrics.

## Data Availability

All data relevant to the study are included in the article.
